# Optogenetic Stimulation Reduces Neuronal Nitric Oxide Synthase Expression After Stroke

**DOI:** 10.1007/s12975-020-00831-y

**Published:** 2020-07-13

**Authors:** Arjun V. Pendharkar, Daniel Smerin, Lorenzo Gonzalez, Eric H. Wang, Sabrina Levy, Stephanie Wang, Shunsuke Ishizaka, Masaki Ito, Haruto Uchino, Terrance Chiang, Michelle Y. Cheng, Gary K. Steinberg

**Affiliations:** Department of Neurosurgery and Stanford Stroke Center, Stanford University School of Medicine, Stanford, CA USA

**Keywords:** Optogenetics, Nitric oxide synthase, Stroke, Functional recovery, Brain stimulation

## Abstract

Post-stroke optogenetic stimulation has been shown to enhance neurovascular coupling and functional recovery. Neuronal nitric oxide synthase (nNOS) has been implicated as a key regulator of the neurovascular response in acute stroke; however, its role in subacute recovery remains unclear. We investigated the expression of nNOS in stroke mice undergoing optogenetic stimulation of the contralesional lateral cerebellar nucleus (cLCN). We also examined the effects of nNOS inhibition on functional recovery using a pharmacological inhibitor targeting nNOS. Optogenetically stimulated stroke mice demonstrated significant improvement on the horizontal rotating beam task at post-stroke days 10 and 14. nNOS mRNA and protein expression was significantly and selectively decreased in the contralesional primary motor cortex (cM1) of cLCN-stimulated mice. The nNOS expression in cM1 was negatively correlated with improved recovery. nNOS inhibitor (ARL 17477)-treated stroke mice exhibited a significant functional improvement in speed at post-stroke day 10, when compared to stroke mice receiving vehicle (saline) only. Our results show that optogenetic stimulation of cLCN and systemic nNOS inhibition both produce functional benefits after stroke, and suggest that nNOS may play a maladaptive role in post-stroke recovery.

## Introduction

Ischemic stroke represents one of the largest contributors to morbidity in the USA [1]. Although significant advances have been made recently with superior clinical outcomes following mechanical thrombectomy for acute stroke, there remains a huge gap in regenerative therapies aimed at promoting subacute post-stroke functional recovery and sensorimotor plasticity [2–4].

Brain stimulation delivered via electrical current or magnetic stimulation represents one emerging therapeutic avenue [5]. However, these techniques non-specifically activate all cell types, making it difficult to elucidate the underlying cell types and mechanisms driving recovery. To circumvent this, our group and others have described the use of optogenetics to selectively activate neurons to promote functional behavioral recovery in mouse stroke models [6–8]. Previously, we demonstrated that optogenetic neuronal stimulation of peri-infarct primary motor cortex (iM1) enhanced functional recovery, with associated increases in levels of neurotrophic factors and plasticity markers. In particular, most of these regenerative factors were upregulated in the contralesional motor cortex (cM1) [8]. We also recently demonstrated that stimulation of the contralesional lateral cerebellar nucleus (cLCN)—a target chosen for its significant excitatory cortical connections—yielded robust functional recovery, as well as increasing plasticity marker GAP43 in the ipsilesional primary somatosensory cortex (iS1) [7]. These findings suggest that targeted brain stimulation can alter molecular changes, which in turn can enhance functional recovery after stroke.

Our previous iM1 optogenetic stimulation study also revealed that stimulated mice exhibited a robust increase in cerebral flow, indicating that repeated neuronal stimulations improved neurovascular coupling responses. Thus, stimulation-induced neuronal excitation and cerebral blood flow may represent one mechanistic link behind functional recovery and plasticity [9]. Various activity-dependent molecules such as ATP, epoxygenase, adenosine, acetylcholine, and COX have been implicated in neurovascular coupling [10]. A key molecular mediator is nitric oxide (NO), a gaseous messenger involved in modulating several physiological functions; these include cerebral blood flow and neurovascular coupling responses, angiogenesis, and neuroplasticity. Nitric oxide is primarily produced by nitric oxide synthase (NOS) [10]. There are three key isoforms of NOS: inducible (iNOS), endothelial (eNOS), and neuronal (nNOS) subtypes. In acute stroke (few days after stroke), eNOS has been shown to be neuroprotective, whereas iNOS and nNOS have been shown to worsen ischemic damage in rodent models [11]. Despite their distinct roles in acute stroke, their role in subacute recovery (days to weeks) remains unclear.

In this study, we investigated the expression of NOS isoforms in optogenetically stimulated mice after stroke. We identified nNOS as the key isoform altered by optogenetic neuronal stimulations and correlated its expression with functional recovery. Furthermore, we interrogated the role of nNOS in subacute recovery using a pharmacological nNOS inhibitor and examined its effects on functional outcome.

## Material and Methods

### Animals

This study utilized Thy1-ChR2-YFP line 18 transgenic male mice (The Jackson Laboratory, Bar Harbor, ME; B6. Cg-Tg(Thy1-COP4/EYFP)18 Gfng/J) and wild-type C57Bl6 male mice (10–12 weeks old). Experimental mice were housed under a 12:12-h light/dark cycle with food and water available ad libitum. All experiments were conducted in compliance with animal care laws and institutional guidelines and approved by the Stanford Institutional Animal Care and Use Committee.

### Stereotaxic Surgery—Optogenetic Cannula Implantation

Optical fibers were implanted into the right cLCN. Mice were first anesthetized with 5% isoflurane and subsequently maintained with 2–3% isoflurane. Body temperature, heart rate, respiration, and mucous membrane color were monitored every 15 min. Mice were secured using ear bars in a digital stereotaxic setup. Artificial tears were applied to prevent corneal damage. An incision was made to the top of the scalp and a small burr hole was drilled on the right side of the skull. The optic fiber cannula (200 μm) was stereotaxically implanted into the cLCN using coordinates from the Paxinos and Franklin’s The Mouse Brain atlas (AP: − 5.80 mm, ML: − 2.25, DV: 2.05). The optical fiber cannula was secured to the skull using C&B Metabond® (Parkell, Inc., Edgewood, NY) and dental cement. Wounds were closed with 5–0 silk suture and 3M™ Vetbond™ tissue Adhesive (3M™, St. Paul, MN). Buprenorphine (0.01 mg/kg) was administered during and post-operation to alleviate pain. Mice were monitored for recovery and returned to their home cages. Mice were randomized and experimenters were blinded. The stereotaxic surgery, stroke surgeries, and behavior tests were performed by three different individuals.

### Transient Middle Cerebral Artery Occlusion

A transient middle cerebral artery occlusion stroke model was utilized. Anesthesia was induced with 5% isoflurane and maintained with 2–3% isoflurane. Body temperature, heart rate, and respiration were monitored every 15 min during surgery to maintain their physiological range. The animals were placed in the supine position and an incision was made at the midline of the neck and the left carotid artery was located. An intraluminal suture of 0.20 mm diameter and 4–5 mm length (70SPRePK5-2045; Doccol Corporation, Sharon, MA) was inserted into the left internal carotid artery and advanced distally to block blood flow to the middle cerebral artery. The suture was left in place for 30 min before being removed. Wounds were closed with sutures and tissue glue. Buprenorphine (0.01 mg/kg) was administered during and post-operation to alleviate pain. For hydration, the animals received daily subcutaneous injections of 0.9% saline for 7 days following the operation and body weights were recorded.

### Stimulation Paradigm

All experimental groups underwent identical behavior handling/training, surgical procedures (stroke and fiber implant in cLCN), and environmental exposures, with the exception that control non-stimulated stroke mice did not receive laser pulses for stimulations. For either stimulation or sham treatment, each mouse was placed in an empty cage after a laser cable was connected to its fiber cannula. During stimulations, the animal was allowed to freely move in the empty cage. In the cLCN-stimulated group, each mouse received one session of stimulation which consisted of three 1-min stimulations separated by 3-min rest intervals. A 473 nm blue laser (OEM Laser Systems, Salt Lake City, UT) was controlled by the Agilent Pulse Generator (AGT33210A; Keysight Technologies, Santa Rosa, CA) and mice were stimulated with a laser set to 10 Hz, 20 msec light pulses with a power range of 0.2–0.4 mW as measured by a power meter (Thorlabs, Newton, NJ). The minimal laser power necessary to elicit movements in the affected forelimb during stimulations was used. Each animal was stimulated at a slightly different power level based on the observation of forelimb movements during each session. Stimulations were performed in the morning between 10 am and noon and behavior tests were performed in the afternoon between 2 and 5 pm, thus allowing for approximately 4 h between stimulations and behavior testing. One session of stimulation was performed on all mice (pre-stroke test stimulation) after the pre-stroke baseline (the day before stroke) in order to evaluate for a positive forelimb movement response during laser stimulation. Lack of response would indicate misplacement of fiber implant or absence of ChR2 gene expression, and these animals were excluded from the study. All mice used in this study exhibited correct functional limb movement response during cLCN stimulations.

### Rotating Beam Test

The rotating beam test is a motor/sensory test to detect neurological deficits after stroke. We measured the speed traveled of mice placed on a rotating white fiberglass beam (length 120 cm, diameter 13 mm, distances marked every cm). The rotating beam (3 rpm) is situated 60 cm above the floor with bubble cushioning covering the floor to reduce the impact from a fall. On a given behavior day, three trials were performed for each mouse and the two highest performance speed values were averaged for data analysis. Behavior testing and optogenetic stimulations were performed by different double-blinded experimenters. Mice were trained on the rotating beam test 3–4 times before the baseline data were recorded. Two baselines were collected: the pre-implant baseline was the day before implantation of the optical fiber and the pre-stroke baseline was the day before stroke surgery. After stroke, the rotating beam test was performed on post-stroke days 4, 7, 10, and 14. Exclusion criteria: (1) mice exhibiting behavioral deficits after implantation, (2) mice without behavioral deficits at day 4 after stroke, and (3) mice without cortical infarcts (validated with histology after sacrifice). Mice included in the study were randomly assigned into two groups (no stim and stim) using an in-house software program that randomly balanced the groups based on two variables: behavior performance at the (1) pre-stroke baseline and (2) post-stroke day 4. Sham mice that had optical fiber implant and sham surgery were used in some studies.

### Photothrombotic Lesion Model

The photothrombotic model can lesion a specific brain region through photo-activation of an injected light-sensitive dye (Rose Bengal) (10 mg/mL; #330000; Sigma-Aldrich, St. Louis, MO) [12], allowing validation of our cLCN implant coordinate. After light illumination, rose bengal is activated and leads to damages in cell membranes of endothelial cells, resulting in interruption of local blood flow and lesioning of the target area. The photosensitive dye rose bengal was made with 0.9% sterile saline. Mice with fiber implant were injected with rose bengal intraperitoneally (8 μl/g). Five minutes after rose bengal injection, the mice were exposed to a green laser (561 nm; OEM Laser Systems, Draper, UT) through the fiber cannula for 10 min (laser power at 1 mW). Mice were returned to cage after photothrombotic model. After 24 h, mice were sacrificed, and brains were stained with MAP2 to stain for brain structures for histological validation of cLCN implant.

### Immunofluorescence Staining

At post-stroke day 15, mice were sacrificed and perfused with cold PBS and 3% paraformaldehyde. Brains were cryoprotected overnight in 20% sucrose/3% PFA solution. After cryoprotection, brains were quickly frozen on dry ice and stored in − 80 °C until sectioning. Brains were sectioned on a cryostat at 30 μm and stored in an antifreeze solution (30% ethylene glycol, 30% glycerol in PBS) at − 20 °C. During immunofluorescence staining, sections were first washed in PBS, then incubated in blocking solution (10% normal goat serum, 1% bovine serum albumin in PBS) for 1 h. Sections were then incubated overnight at 4 °C in primary antibody diluted in the blocking solution (MAP2; Cell Signaling Technology®, Danvers, MA; #8707; dilution 1:200 and CD68 Abcam ab53444, dilution 1:200). Next day, sections were washed in 0.3% PBS-triton X and incubated for 2 h at room temperature with 1:500 secondary antibody (Alexa Fluor™ 488 or 594; Invitrogen, Carlsbad, CA) diluted in the blocking solution. DAPI (1:2000) was added during the last 5 min of the secondary antibody incubation. Sections were then washed in PBS, mounted, and cover-slipped. High-resolution-tiled images were acquired using a Zeiss confocal LSM 800 microscope (Zeiss, Oberkochen, Germany) with Zeiss ZEN software.

### Infarct Quantitation

Infarct lesion area was identified by CD68-positive-activated monocytes/macrophages. CD68-positive areas were confirmed to match the MAP2-negative areas (neuronal loss). Infarct size was analyzed using the National Institutes of Health (NIH) ImageJ software (https://imagej.nih.gov/ij/) [13].The percentage of infarct area per whole hemisphere was calculated as % infarct area by this formula: 100 × [total contralesional hemisphere area – (ipsilesional intact area)]/(total contralesional hemisphere area). This infarct calculation corrects for any edema or atrophy in the brain. The analyses were performed on coronal sections at the striatal level (range: 0.5 mm to 1.2 mm anterior to Bregma), and the hippocampal level (range: 1.3 mm to 2.1 mm posterior to Bregma) [14].

### RNA Extraction and Quantitative PCR

Experimental animals were sacrificed on post-stroke day 15 and perfused with cold sterile × 1 PBS; brain regions (iM1, cM1) were dissected on ice with × 1 PBS. Dissected regions were kept on ice in RNAlater and frozen at − 80 °C. RNA was extracted with the Qiagen RNeasy Plus kit (QIAGEN, Germantown, MD). First-strand cDNA synthesis was performed using SuperScript Reverse Transcriptase II with oligo dT12-18 primer (Invitrogen). qPCR was performed using the CFX96 Real-Time PCR Detection system (Bio-Rad, Hercules, CA). qPCR reaction mixtures were prepared using Taq polymerase (#4369016; Life Technologies, Carlsbad, CA) and Taqman primers targeting mouse NOS isoforms (NOS1 Mm00435175_m1, NOS2 Mm00440502_m1, NOS3 Mm00435217_m1; Life Technologies, Carlsbad, CA). qPCR data were analyzed using the delta delta CT method.

### Western Blot Analysis

Western blot analysis was carried out on extracted tissue samples to verify the downregulation of nNOS expression at the protein level. Twenty microliters of each sample (1 μg/ul) was loaded into a precast Bio-Rad Criterion TGX 4–15% gel in a × 1 Bio-Rad Tris/Glycine/SDS buffer and run at a constant 200 V for 45 min using a Bio-Rad PowerPac Universal power supply. Protein was transferred from the precast Criterion TGX 4–15% gel onto a PVDF membrane using a Bio-Rad TransBlot Turbo transfer system. Membranes were blocked for 1 h at room temperature using a 5% solution of Bio-Rad Blotting-Grade Blocker (#1706404) in × 1 phosphate-buffered saline solution (PBS). Membranes were first incubated with primary antibody overnight at 4 °C using a 1:1000 dilution of rabbit polyclonal anti-nNOS antibody (sc-648; lot D0915; Santa Cruz Biotechnology, Dallas, TX) in 5% Blotting-Grade Blocker solution, followed by incubation with secondary antibody for 1 h at room temperature using a 1:5000 dilution of Cell Signaling Technology® anti-rabbit HRP-linked antibody (7074S) in 5% Blotting-Grade Blocker solution. Membranes were developed using a GE Healthcare ECL Prime Western Blotting Detection Reagent system (RPN2232). Membranes were visualized using a Bio-Rad ChemiDoc MP imaging system and data was quantified using NIH ImageJ software [13].

### nNOS Inhibitor Delivery

Intraperitoneal injections were administered each morning on post-stroke days 5–14 using 1 mL Leur-Lok plastic disposable syringes (Fisher Scientific, Pittsburgh, PA). The nNOS inhibitor ARL 17477 dihydrochloride (#3319, N-[4-[2-[[(3-Chlorophenyl)methyl]amino]ethyl]phenyl]-2-thiophenecarboxamide dihydrochloride, ≥ 99% purity via HPLC, Tocris BioScience, Minneapolis, MN) was prepared by dissolving in 0.9% sterile saline. Mice in the experimental groups received injections of the nNOS inhibitor at 10 mg/kg and control animals received injections of 0.9% saline in equal volume.

### Statistical Analysis

All statistics were performed using GraphPad Prism (version 5.00, GraphPad Software, La Jolla California USA, www.graphpad.com). Behavioral results were analyzed with two-way ANOVA followed by Bonferroni’s post hoc test. The qPCR mRNA expression data were assayed via one-way ANOVA with Fisher’s least significant difference (LSD) test. The Western blot data were analyzed using a two-tailed Student’s *t* test.

## Results

### Optogenetic Stimulation of cLCN Promotes Functional Recovery After Stroke

**Fig. 1:**
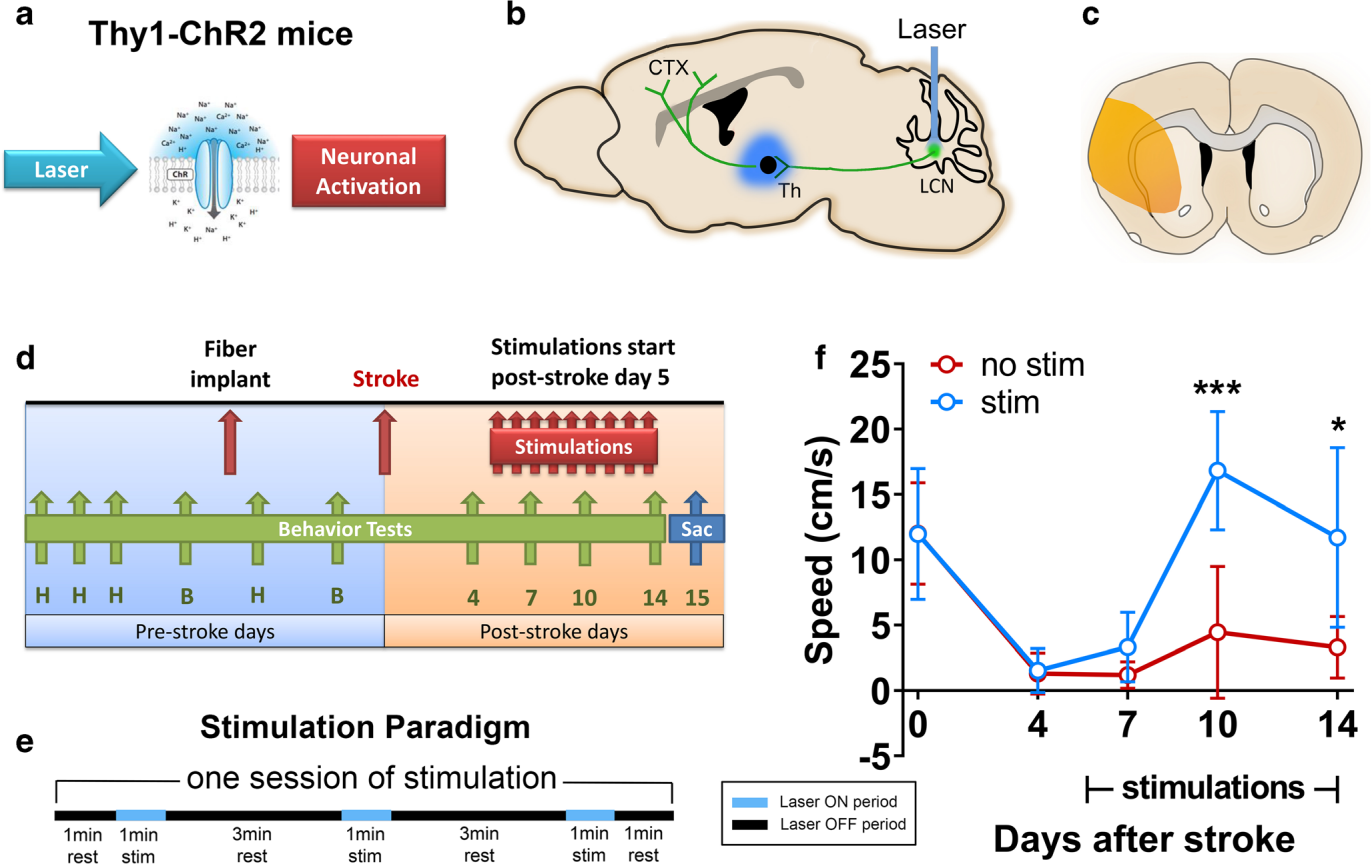
Optogenetic neuronal stimulation of the cLCN promotes behavioral recovery after stroke. **a** Thy1 mice-expressing channelrhodopsin 2 (ChR2) were used in this study. Blue laser stimulation activates ChR2 and causes neuronal excitation. **b** Schematic depicting the optogenetic laser stimulation site (blue bar) in the cLCN. Efferent projections travel through the superior cerebellar peduncle, decussate in the midbrain tegmentum and terminate in the ipsilesional ventrolateral thalamus (blue). Second-order neurons then project to multiple cortical regions including prefrontal, premotor, motor, and posterior parietal cortex. **c** Schematic illustrating the cortical and striatal infarction produced by transient middle cerebral artery occlusion. **d** Experimental paradigm. Mice were pre-trained (*H*) on the rotating beam test prior to the pre-fiber implant baseline (*b*) and the pre-stroke baseline data (day 0). Each mouse in the treatment group received one session of stimulations daily, from post-stroke day 5 and continued until day 14. Behavior tests were performed at post-stroke days 4, 7, 10, and 14. Mice were sacrificed at day 15 for qPCR and Western blot analysis. **e** Optogenetic neuronal stimulation paradigm. Each stimulation session consists of three 1-min stimulations with 3-min rest periods in between. Laser ON periods (blue) and laser OFF periods (black) are indicated. **f** Repeated neuronal stimulations of cLCN produced post-stroke recovery. cLCN-stimulated mice demonstrated significant improvement in speed (cm/s) on the rotating beam task at 10 and 14 days post-stroke. *n* = 4 for stroke + no stim, *n* = 4 for stroke + stim. Two-way ANOVA with Bonferroni’s post hoc test, *P < 0.05, ***P < 0.001. Data are expressed as mean ± SD

**Fig. 2:**
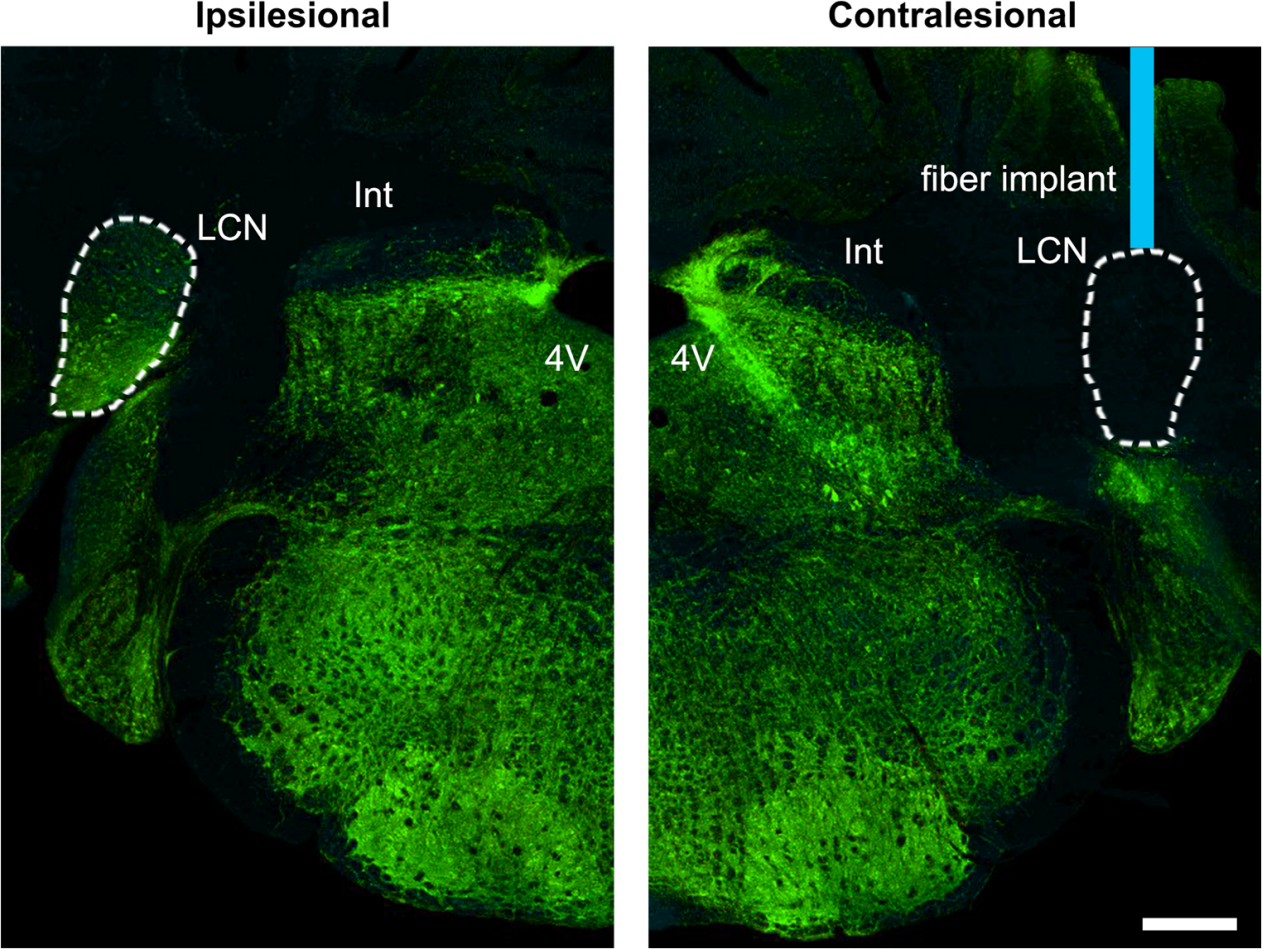
Validation of cLCN targeting using photothrombotic lesion model. Representative images depict LCN in the ipsilesional and contralesional cerebellar coronal sections (dotted line outlines the location of LCN). Brain structures were visualized by immunostaining with neuronal marker MAP2 (green). Note that LCN on the ipsilesional side is intact, while LCN on the contralesional side where the fiber was implanted (blue) has been lesioned after the photothrombotic model. Int, interposed nuclei; LCN, lateral cerebellar nuclei; 4 , 4th ventricle. Scale bar = 500 μm

We first evaluated whether cLCN optogenetic stimulation would produce a functional benefit after stroke. Stimulation of cLCN leads to activation of the dentate-thalamo-cortical pathway (Fig. 1b). Transgenic Thy1-ChR2-YFP mice underwent handling and baseline behavioral testing prior to optogenetic cannula implantation and prior to transient middle cerebral artery occlusion (Fig. 1d). Neuronal optogenetic stimulation was delivered on post-stroke days 5–14 for mice in the treatment group. Each stimulation session consisted of three 1-min stimulations (10 hz, 20 ms, 473 nm) with 3-min rest periods in between (Fig. 1e). cLCN-stimulated stroke mice demonstrated significant improvement in speed (cm/s) on the rotating beam task at 10 and 14 days post-stroke (Fig. 1f). All mice included in the study exhibited functional limb movements during test stimulations, indicating proper placement of the fiber in cLCN. Furthermore, we demonstrated correct fiber placement in cLCN using a photothrombotic lesion model (Fig. 2).

### Optogenetic Stimulation of cLCN Selectively Decreases nNOS mRNA Expression in cM1

**Fig. 3:**
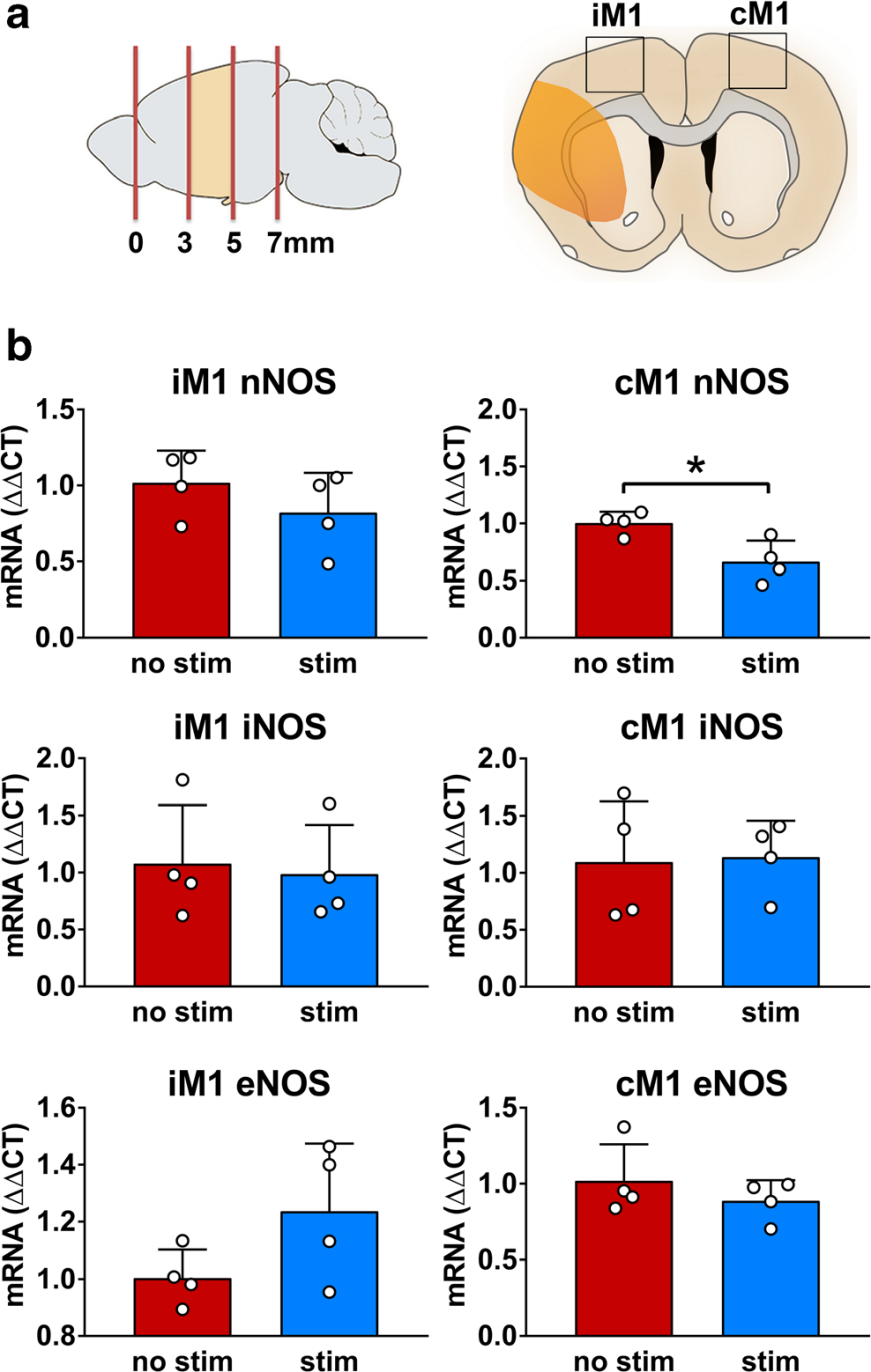
nNOS mRNA is selectively decreased in cM1 after optogenetic stimulation of the cLCN. **a** Schematic depicting the location of iM1 and cM1 within tissue sections selected for RNA and protein analysis. mRNA expression of NOS isoforms (nNOS, iNOS, eNOS) was quantified in iM1 and cM1 of cLCN-stimulated and non-stimulated mice at post-stroke day 15. **b** Bar graphs with scatter dot plot shows nNOS mRNA expression was significantly decreased in cM1 of cLCN-stimulated animals. Data are expressed as mean ± SD. There was no significant difference in nNOS mRNA expression in iM1 between stimulated and non-stimulated mice. Similarly, there were no differences in iNOS or eNOS between groups in iM1 or cM1. *n* = 4 for each group. *P < 0.05, significant difference between stimulated and non-stimulated stroke mice, one-way ANOVA with Fisher’s LSD

We next sought to investigate the mRNA expression of the key NOS isoforms in stimulated mice exhibiting improved recovery compared to non-stimulated stroke mice. Quantitative PCR was used to measure the mRNA expression of NOS isoforms (nNOS, iNOS, eNOS) in iM1 and cM1 of cLCN-stimulated and non-stimulated mice at post-stroke day 15. nNOS mRNA expression was significantly decreased in cM1 of cLCN-stimulated animals (Fig. 3). nNOS mRNA expression in iM1 was not significantly different between stimulated- and non-stimulated mice. Similarly, there were no differences in iNOS or eNOS between groups in iM1 or cM1.

### nNOS mRNA Expression in cM1 are Negatively Correlated with Behavioral Recovery

**Fig. 4:**
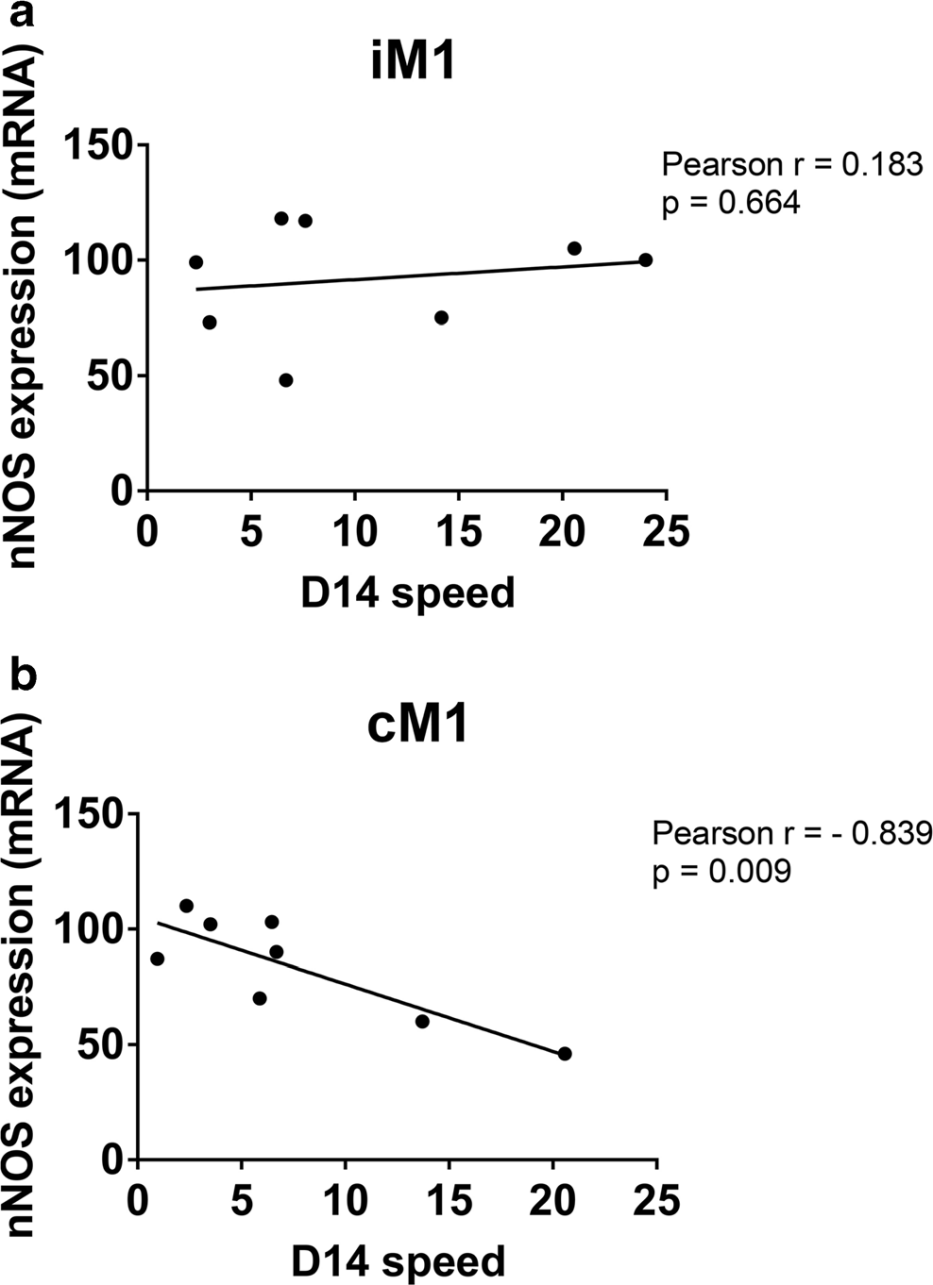
nNOS mRNA expression in cM1 is negatively correlated with behavioral recovery. Correlation analysis between speed (cm/s) across the rotating beam at post-stroke day 14 and nNOS mRNA in **a** iM1 and **b** cM. Lower nNOS mRNA levels in cM1 correlated with increased speed (*n* = 8; Pearson *r* = − 0.839; *p* = 0.0092). There was no correlation between iM1 nNOS mRNA expression and speed in the rotating beam

To determine whether the nNOS expression was correlated with functional recovery observed after optogenetic cLCN stimulation after stroke, we plotted speed (cm/s) across the rotating beam at post-stroke day 14 against iM1 and cM1 mRNA expression for all experimental animals (Fig. 4). Lower nNOS mRNA levels in cM1 significantly correlated with improved performance in speed (*n* = 8; Pearson *r* = − 0.839; *p* = 0.0092). There was no correlation between iM1 nNOS mRNA expression and speed in the rotating beam test (*n* = 8; Pearson *r* = 0.183; *p* = 0.6644).

### Optogenetic Stimulation of cLCN-Reduced nNOS Protein Expression

**Fig. 5:**
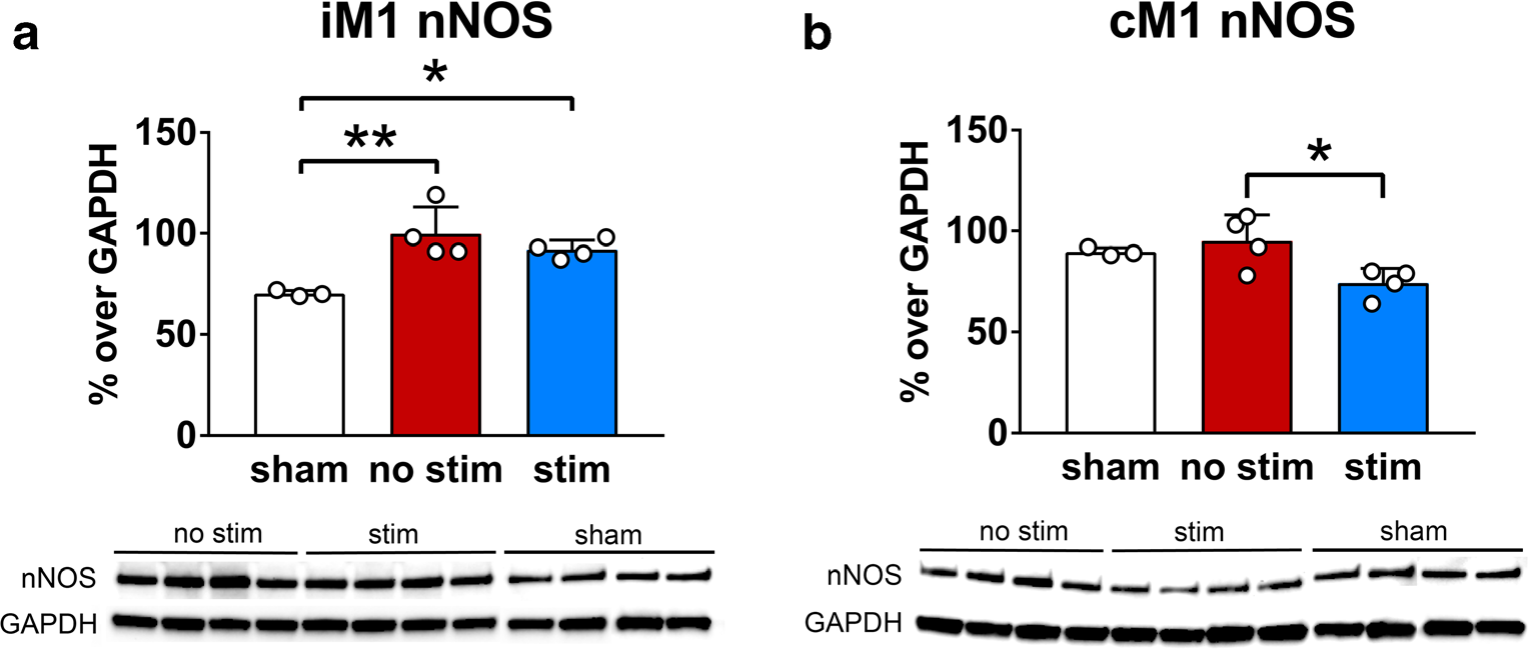
Optogenetic stimulation of the cLCN decreases nNOS protein expression in cM1. Bar graphs with scatter dot plot shows the results of western blot analysis in **a** iM1 and **b** cM1 mice brain sacrificed on post-stroke day 15. Data are expressed as mean ± SD. **a** In iM1, both cLCN-stimulated and non-stimulated mice demonstrated an increase in nNOS protein levels compared to sham animals. There was no difference in nNOS protein expression between stimulated or non-stimulated mice. **b** In cM1, cLCN-stimulated mice exhibited a decreased nNOS protein level compared to non-stimulated animals. There was no difference in nNOS protein levels between non-stimulated stroke animals and sham mice. nNOS protein levels are expressed as percentage optimal density measurement over GAPDH. *n* = 4 for each group. *P < 0.05; significant difference between stimulated and non-stimulated groups, Student’s *t* test

To determine whether this difference in nNOS mRNA expression was recapitulated at the protein level, Western blot analysis was performed on iM1 and cM1 brain samples from mice sacrificed on post-stroke day 15. In iM1, both stimulated and non-stimulated animals demonstrated increased nNOS protein levels as compared to sham animals (Fig. 5). However, there was no difference between stimulated and non-stimulated stroke groups. In cM1, cLCN-stimulated mice exhibited decreased nNOS protein expression when compared to non-stimulated mice (Fig. 5).

### Systemic nNOS Inhibition Produces Moderate Functional Recovery After Stroke

**Fig. 6:**
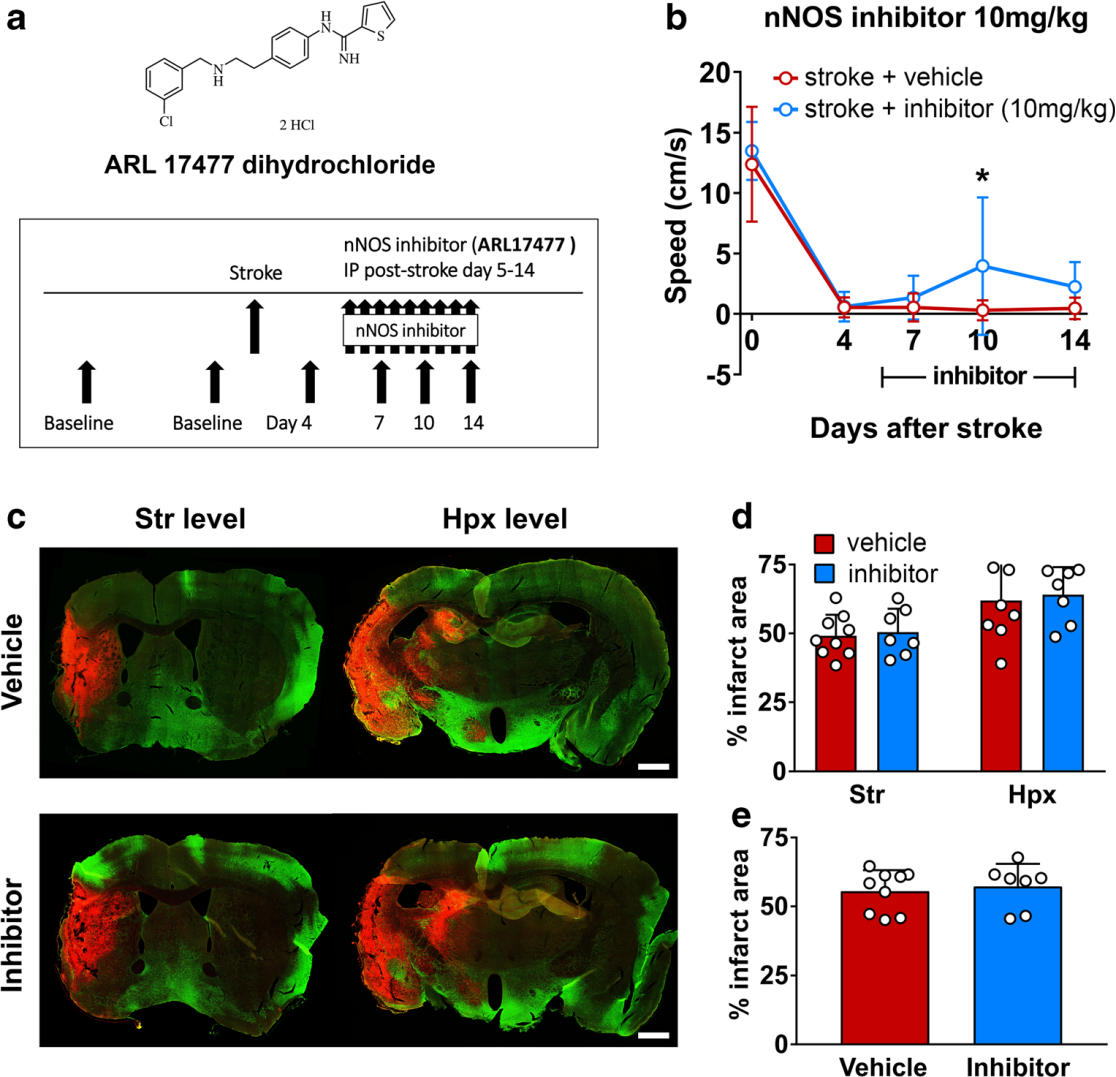
Systemic nNOS inhibition produces behavioral recovery after stroke. **a** Molecular structure of ARL 17477 dihydrochloride which was used for the systemic nNOS inhibition studies. Experimental paradigm depicting mice undergoing baseline behavioral testing followed by transient middle cerebral artery occlusion. Mice received intraperitoneal vehicle (saline) or 10 mg/kg nNOS inhibitor (ARL 17477 dihydrochloride) on post-stroke days 5–14. Behavior on the rotating beam test was performed on post-stroke days 7, 10, and 14. **b** Mice receiving nNOS inhibition after stroke experienced a significant improvement over vehicle treated animals at post-stroke day 10. Data are expressed as mean ± SD. *n* = 9 for stroke + vehicle; *n* = 7 for stroke + nNOS inhibitor; two-way ANOVA with Bonferroni’s post hoc test, *P < 0.05. **c** Representative coronal brain sections at striatum (Str) and hippocampus (Hpx) level in vehicle and inhibitor groups. The infarct area was identified by CD68-positive activated monocytes/macrophages (red) and neuronal MAP2-negative areas (green). Scale bar = 1 mm. Bar graphs with scatter dot plot show that **d** % infarct area at the Str and Hpx level and **e** total infarct area are similar between vehicle and inhibitor group. *n* = 9 for vehicle group, *n* = 7 for inhibitor group

Since optogenetic cLCN stimulation decreased nNOS expression in cM1 after stroke and this reduced nNOS level was correlated with functional recovery, we aimed to interrogate the role of nNOS in subacute recovery phase using a small molecule nNOS inhibitor (ARL 17477 dihydrochloride) (Fig. 6a). ARL 17477 was delivered systemically (i.p.) on post-stroke days 5–14 in a treatment paradigm similar to the optogenetic stimulations. In contrast to stroke mice receiving intraperitoneal vehicle (saline) only, mice receiving the nNOS inhibitor after stroke exhibited a significant functional improvement at post-stroke day 10 on the rotating beam test **(**Fig. 6b**)**.

Furthermore, we investigated whether nNOS inhibitor alters infarct size. Figure 6 c shows representative coronal sections from the vehicle and the nNOS inhibitor-treated mice (striatal and hippocampal level). Infarct size was visualized by double staining with CD68 (marker for activated monocytes/macrophages) and MAP2 (pan-neuronal marker). Quantification of infarct showed that nNOS inhibitor did not alter infarct area (Fig. 6d, e).

## Discussion

We have shown that optogenetic stimulation of cLCN in a mouse stroke model selectively decreases nNOS expression and the observed reduction is correlated with improved recovery. Interestingly, selective nNOS reduction was observed in cM1 and not iM1 (Figs. 3 and 5), highlighting the involvement of cM1 in stimulation-induced changes in nNOS expression. We further investigated the role of nNOS in post-stroke recovery using a pharmacological nNOS inhibitor. nNOS inhibitor-treated stroke mice exhibited a significant functional improvement when compared to stroke mice receiving vehicle (saline) only (Fig. 6). Taken together, our results suggest that nNOS may play a maladaptive role in subacute post-stroke recovery. Thus, neuronal stimulations and/or pharmacologic inhibition targeting nNOS may yield promising functional benefits in stroke.

It is interesting that optogenetic stimulation selectively reduced nNOS, but not iNOS or eNOS, mRNA levels. Previous studies have characterized nNOS as a key mediator of angiogenesis and neuroplasticity and described nNOS as a key element in excitotoxicity [15]. eNOS has previously been shown to be neuroprotective following ischemic stroke [16–18], whereas nNOS and iNOS have been shown to worsen ischemic injury in the acute timeframe (few days after stroke) [11]. nNOS overexpression has been implicated as a maladaptive process in acute stroke. For example, Khan and colleagues have parsed out the NO metabolome pathway and described detrimental downstream effectors of nNOS including peroxynitrite and the AMP-kinase pathway [19]. In their study, they successfully blocked peroxynitrite and AMP-kinase signaling using S-nitrosoglutathione (GSNO) in the acute setting and demonstrated reduced neuronal injury in a rat model of reperfusion ischemia. We expand their work, and others, into the subacute recovery phase (days to weeks) of stroke and demonstrated that nNOS may also be maladaptive during recovery, as reduced nNOS expression after optogenetic stimulation is significantly correlated with improved recovery (Fig. 4). Furthermore, pharmacological inhibition of nNOS improved functional recovery (Fig. 6).

Unlike acute stroke, there is a dearth of literature characterizing the role of nNOS in subacute stroke. In a single report, Zhou and colleagues disrupted the N-methyl-d-aspartate receptor (NMDAR)–postsynaptic density protein-95 (PSD-95) interaction using Tat-NR2B9c given PD4 onwards [20]. Pharmacologic treatment successfully improved sensorimotor and spatial learning/memory ability. The key implication for nNOS in subacute recovery is that PSD-95 is a scaffold protein that links NMDAR and nNOS at excitatory synapses. In fact, in their study, NOS −/− animals did not exhibit therapeutic benefit from Tat-NR2B9c. Therefore, disruption of this complex potentially links nNOS to excitatory brain stimulation in our experiments. Indeed, the constellation of peri-ischemic neuronal stimulation, increased neurotrophic, and plasticity markers as well as improvements in CBF may be mediated at the synaptic level by nNOS as one of the master regulators driving subacute recovery.

Several other groups have utilized ARL 17477 to inhibit nNOS in acute stroke studies. Zhang and colleagues carried out a similar study in rats utilizing the same nNOS inhibitor, ARL 17477, to successfully reduce infarct size [21]. In their study, pharmacological nNOS inhibition successfully reduced ischemic damage in acute stroke at the 10 mg/kg, 3 mg/kg, and 1 mg/kg levels. Likewise, O’Neil and colleagues reported administration of ARL17477 during acute stroke to be neuroprotective in both rats and gerbils [22]. In addition, the same group also performed pharmacokinetic studies with this ARL 17477 nNOS inhibitor and demonstrated that this compound crosses the blood-brain barrier [22]. One drawback to our study includes the modest results from systemic pharmacologic inhibition, where we showed that nNOS-treated mice exhibited improved recovery at post-stroke day 10 (Fig. 6). This is likely due to several reasons: (1) the systemic pharmacological delivery (intraperitoneally) which would inhibit nNOS globally and (2) the repeated administration of ARL17477 from day 5 to 14 may have some inhibition effects on eNOS as well, since the IC50 values for ARL17477 are 1 and 17 μM for nNOS and endothelial NOS, respectively. Our findings are promising, but limited and should be followed up with further studies using local and selective inhibition of nNOS via pharmacological or genetic approaches. This point is further highlighted by a recent report by Kleinshnitz questioning the efficacy of PSD-95 inhibitors [23]. Their study confirmed the deleterious role of nNOS, but failed to replicate the positive results of PSD-95 inhibitors outlined earlier. Similarly, it is possible that the maladaptive post-stroke effects of nNOS are divergent and involve multiple pathways. Ni and colleagues have reported a related, but distinct interaction with carboxy-terminal postsynaptic density-95/discs large/zona occludens-1 ligand of nNOS (CAPON) which interacts with nNOS [24]. They found that inhibition of the nNOS-CAPON interaction can facilitate dendritic remodeling and synaptic plasticity in addition to behavioral benefit in rodent models.

It is clear that subacute neuronal stimulation after stroke can produce some degree of plasticity, and the constellation of excitatory synapses, changes in CBF and key regulators like nNOS contribute to functional recovery. The effect of cLCN stimulation on nNOS expression in cM1 is likely through a secondary effect from iM1 to cM1 through transcallosal fibers. nNOS expression has been shown to be in both excitatory and inhibitory neurons [25, 26]. Future studies identifying the subcellular distribution of nNOS will further elucidate the molecular and circuit mechanisms of cLCN stimulation-induced recovery. As the mechanistic details continue to be elucidated, novel downstream targets can be identified to inform new clinical therapies for stroke patients.
